# Electronic health record access by patients as an indicator of information seeking and sharing for cardiovascular health promotion in social networks: Secondary analysis of a randomized clinical trial

**DOI:** 10.1016/j.pmedr.2018.12.011

**Published:** 2019-01-14

**Authors:** Sherry-Ann N. Brown, Hayan Jouni, Iftikhar J. Kullo

**Affiliations:** Department of Cardiovascular Diseases, Mayo Clinic, Rochester, MN, United States of America

**Keywords:** Genetics, Risk factors, Risk assessment, Behavior modification, Electronic health records, Personal health records, Patient portals, Patient engagement, Social network

## Abstract

We investigated electronic health record (EHR) access as an indicator of cardiovascular health promotion by patients in their social networks, by identifying individuals who viewed their coronary heart disease (CHD) risk information in the EHR and shared this information in their social networks among various spheres of influence. In a secondary analysis of the Myocardial Infarction Genes trial, Olmsted County MN residents (2013–2015; n = 203; whites, ages 45–65 years) at intermediate CHD risk were randomized to receive their conventional risk score (CRS; based on traditional risk factors) alone or also their genetic risk score (GRS; based on 28 genomic variants). We assessed self-reported and objectively quantified EHR access via a patient portal at three and six months after risk disclosure, and determined whether this differed by GRS disclosure. Data were analyzed using logistic regression and adjusted for sociodemographic characteristics, family history, and baseline CRS/GRS. Self-reported EHR access to view CHD risk information was associated with a high frequency of objectively quantified EHR access (71(10) versus 37(5) logins; *P* = 0.0025) and a high likelihood of encouraging others to be screened for their CHD risk (OR 2.936, CI 1.443–5.973, *P* = 0.0030), compared to the absence of self-reported EHR access to view CHD risk information. We thereby used EHR access trends to identify individuals who may function as disseminators of CHD risk information in social networks, compared to individuals on the periphery of their social networks who did not exhibit this behavior. Partnering with such individuals could amplify CHD health promotion.

**Clinical Trial Registration**: Myocardial Infarction Genes (MI-GENES) Study, NCT01936675, https://clinicaltrials.gov/ct2/show/NCT01936675.

## 1 Introduction

Much of the health information stored in the EHR can be accessed by patients via a patient portal. In one study, the leading reason (reported by over 7 in 10 individuals) for accessing one's EHR was to monitor one's health (e.g., to obtain test results) (Patel et al., 2014). The second leading reason (reported by over 4 in 10 individuals) for accessing the EHR was to share the health information with others (e.g., health care provider, family member). In another study of EHR access by patients, the majority of participants (>6 in 10 individuals) wanted to grant access to their test results and other health information to their family members, friends, and neighbors (Zulman et al., 2011). This suggests that EHR access by individuals may play a role in health promotion in their non-digital social and health networks. However, whether the degree of EHR access is associated with sharing health information that can facilitate health promotion in social networks is unknown. Further, whether EHR access could identify individuals who are more likely to share health information in their social networks has not been explored.

Such sharing of health information may promote adoption of preventive measures, particularly for common chronic diseases, in social networks. Furthermore, such adoption may be amplified when risk estimates include genetic susceptibility variants. Multi-locus genetic risk scores for CHD, for example, refine disease risk (Ganna et al., 2013; Tikkanen et al., 2013; Hughes et al., 2012; Ripatti et al., 2010; Iribarren et al., 2016; Khera et al., 2016) and predict CHD events and clinical benefit from statins (Mega et al., 2015), and genetic risk disclosure to patients has been shown to influence medication use (Kullo et al., 2016). EHR access to obtain one's CHD risk information and subsequent sharing of this information could potentially influence health promotion in non-digital social networks. While other non-traditional CHD risk factors could possibly also influence information exchange in non-digital social networks, sharing of GRS disclosure among family members with shared familial risk (and others in social networks) could encourage genetic testing particularly among family members and others in their biological social networks.

Prior studies of EHR access largely centered on patient's perspectives on the importance and security of having access to their EHR, as well as the details of why and how patients use the EHR, and with whom patients share data from their EHR (Patel et al., 2014; Zulman et al., 2011; Ammenwerth et al., 2017; de Lusignan et al., 2014). Some researchers are starting to further analyze EHR access, e.g., with social network analyses to determine individual patients' network of health care professionals (Zhu et al., 2016). However, there is no established methodology for quantifying EHR access to identify individuals who may share health information in their social networks. Some institutions and hospitals enable patient access to EHR data through websites or mobile applications. In such settings, objective quantification of EHR access may be more feasible and could become the gold standard for measuring EHR access. When such monitoring of EHR access is not feasible, self-reports of EHR access should be considered. Further, self-reports of EHR access on surveys allow individuals to elaborate their reasons for accessing the EHR (Ammenwerth et al., 2017; de Lusignan et al., 2014) (e.g., to retrieve their CHD risk information, and so on), which are not captured by objective quantification of overall EHR access.

In this secondary analysis of the Myocardial Infarction Genes (MI-GENES) trial, we hypothesized that individuals who self-reported EHR access to seek their CHD risk information actually accessed the EHR frequently and shared the gleaned health information widely in their social networks. We then developed a conceptual model of the role of these individuals in dissemination of health information in their social networks.

## 2 Methods

### 2.1 The MI-GENES study

**Fig. 1:**
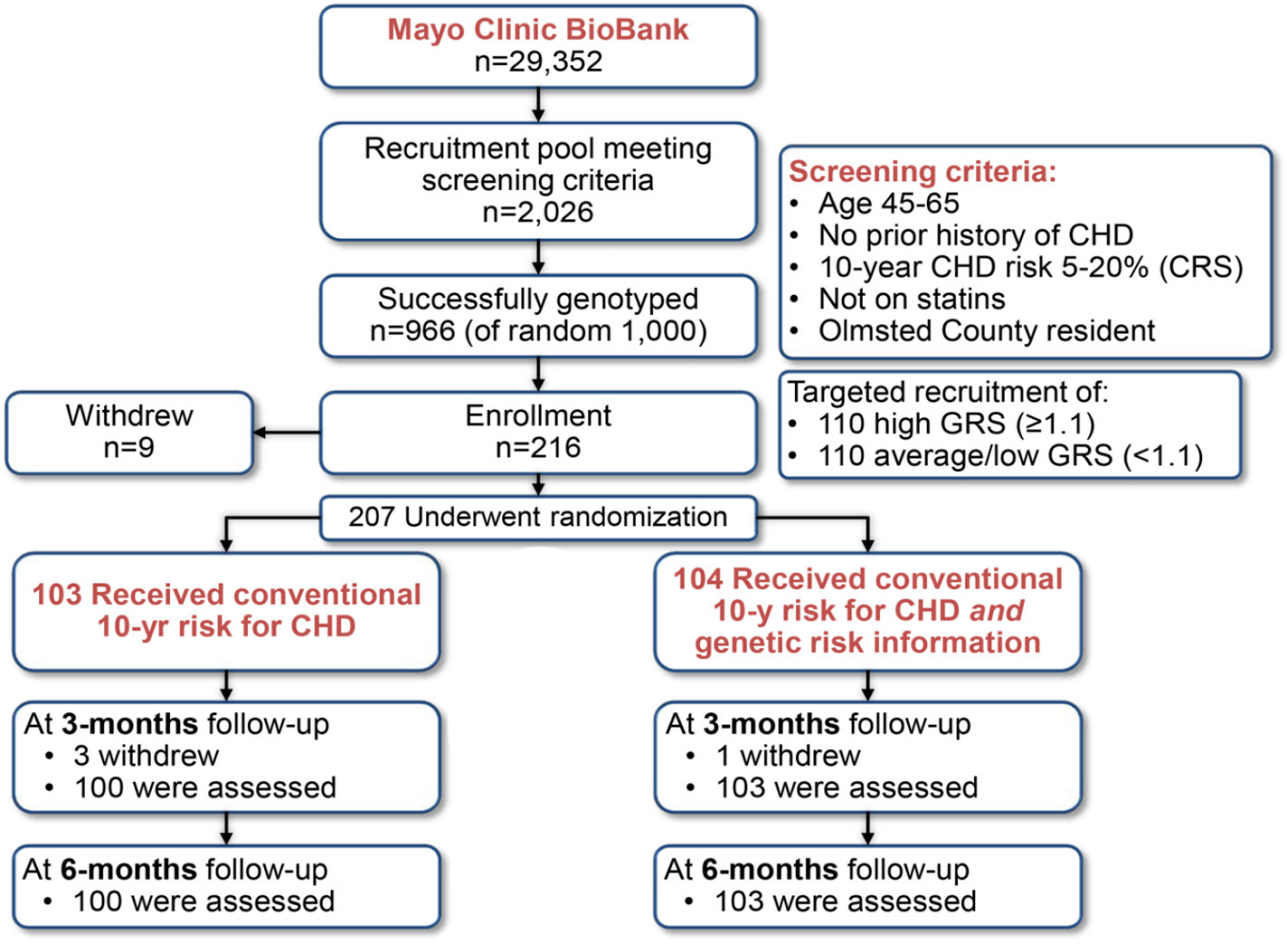
The MI-GENES study design. Approximately 2000 of 30,000 individuals available in the Mayo Clinic BioBank met the screening criteria for our study (ages 45–65 years, with no history of CHD or statin use and at intermediate risk for CHD; Olmsted County, MN; 2013–2015). A random 1000 individuals were selected from among the 2000. Of these, 966 individuals were successfully genotyped. Targeted recruitment of at least 110 individuals with high GRS and 110 with average/low GRS led to enrolment of 216 participants; 9 withdrew. Ultimately, 207 individuals were randomized to the CRS group to receive their CRS alone, or the GRS group to also receive a GRS; 4 withdrew and 203 remained at follow-up. Surveys for EHR access and Internet Use were completed at baseline and at 3 months post-disclosure (internet use outside of the patient portal only) and 6 months post-disclosure (internet use outside of the patient portal as well as for patient portal access). Surveys for information sharing and social network were completed at three and six months post-disclosure. CHD = coronary heart disease; CRS = conventional risk score; GRS = genetic risk score.

In the MI-GENES study (Kullo et al., 2016; Kullo et al., 2015) (Fig. 1), participants gave written informed consent, and the Mayo Clinic Institutional Review Board approved the study. Participants (n = 203) were white 45–65 year old residents in Olmsted County, Minnesota, with no prior history of statin therapy or CHD, with an intermediate range 10-year CHD risk (5%–20%) based on the Framingham risk score (National Cholesterol Education Program ATP III Guidelines At-A-Glance, 2001) (conventional risk score; CRS). Patients received only their CRS (‘CRS participants’) or also a 28-variant GRS (‘GRS participants’), using standardized statements and an electronic decision aid (Kullo et al., 2016). The GRS (minimum 0.5, maximum 2.0; Appendix Fig. A.1) was stratified as high GRS (≥1.1, i.e., ≥10% increase in CHD risk relative to the general population) or low GRS (<1.1) and disclosed inperson and then placed in the EHR. Blood samples were obtained to determine LDL-C levels in response to initiation of statin therapy in the two randomized groups, as the primary endpoint of the study (Kullo et al., 2016). Self-reported information seeking and sharing behaviors and objective quantification of total EHR access were used to identify individuals who may disseminate CHD risk information, as a secondary endpoint. In addition, a score was developed to estimate the extent of information sharing by these individuals in their social networks.

### 2.2 Patient-administered surveys

Participants' recall of their disclosed CHD risk was assessed at three and six months after risk disclosure using the question, “Out of 100 people like you, how many individuals would have a heart attack over the next 10 years?”

In the EHR access survey administered three and six months after risk disclosure (Appendix Table A.1), questions regarding communicating with a doctor's office and keeping track of personal health information were adapted from the National Cancer Institute's Health Information National Trends Surveys (HINTS; http://hints.cancer.gov) from 2008 and 2005, respectively. Questions about signing up for access and using the portal to obtain their CHD risk information were added to identify individuals who access the portal to review their CHD risk.

In the Internet Use survey (Appendix Table A.2), questions regarding seeking information outside of the EHR about how personal health habits and genetics affect CHD risk were adapted from Kaphingst et al. (2012) and HINTS 2005, and were administered three and six months post-disclosure. The following questions were administered at baseline and three and six months post-disclosure. Questions about internet use for information about CHD and other medical conditions were adapted from HINTS 2008 and 2012, respectively. Questions about internet use for help with lifestyle modification or other health needs were adapted from HINTS 2011 and 2005, respectively.

The ‘Information Sharing’ survey was completed at three and six months post-disclosure (Appendix Table A.3). A question regarding discussing CHD risk with others was adapted from Kaphingst et al. (2012), and a question about encouraging others to be screened for their CHD risk was added specifically to assess the potential for health promotion in social networks. A question about discussing CHD risk with friends, family members, co-workers, or others (including primary care provider (PCP)) was also adapted from Kaphingst et al. (2012). Each of four spheres of influence in non-digital social networks was given one point: family, friends, co-workers, and others (e.g., primary care provider), with the sum termed the ‘information sharing radius’. A question about sharing CHD risk with parents, siblings, spouse, children, extended family, or the PCP was added to expand on the previous question.

For the ‘Social Network’ survey administered at baseline and at three and six months post-disclosure (Appendix Table A.3), questions about having friends or family with whom they discussed health information or having community organizations that provided health information were adapted from the HINTS 2005 survey.

### 2.3 Quantification of EHR access

Historical data from the medical record were used to quantify frequency of EHR access over the course of the study. The actual number of discrete instances in which each participant's personal, private, and unique username and password login credentials were manually entered on the internet website or in the Mayo Clinic mobile patient application to access the patient's EHR, from the initial visit until at least 12 months post-disclosure (with an average of 14 months post-disclosure), was termed ‘TotalLogin’ (‘Expansion of Results’ in Appendix C) (Appendix Tables A.4–7). Associations between TotalLogin and favorable survey question responses were determined using linear regression (Appendix Table A.4).

### 2.4 Conceptual model

We developed a conceptual model of the role of individuals who may extensively share health information in social networks. Connecting lines were used to represent EHR access via a patient portal by study participants. In studies of social networks, the quantified frequency of accessing health information is represented by the width of the connecting line (or ‘tie’) between an individual and the source of that information (Zhu et al., 2016), and captures the “strength” of that information seeking or communication tie. Accordingly, more frequent access of the EHR in our study was represented by a wider connecting line, consistent with a stronger information seeking relationship than represented by narrow connecting lines. In addition, we examined the formation of social or communication links or ties – connections between patients and the four pre-specified spheres of influence in non-digital social networks (family, friends, co-workers, and others such as primary care provider). The report of sharing health information with a particular sphere of influence was captured as formation of a communication tie with that sphere of influence. We did not examine links between patients and individual recipients of information shared by the patient. In this way, we used communication ties with the evaluated spheres of influence to build a simple patient-centered information sharing network.

### 2.5 Statistical methods

Survey data were stored using the Research Electronic Data Capture (REDCap) (Harris et al., 2009) software. Data were analyzed in JMP V9.0.2 (SAS Institute Inc., Cary, NC). We reported scores for individual statements; a higher score indicated a more favorable response. Each question used a binary scale, with 1 for “no” (unfavorable) and 2 for “yes” (favorable). Logistic regression was used to estimate the randomized group effects on survey responses. Descriptive statistics were used for baseline sociodemographic characteristics: age, sex, family history of CHD, and level of education. All survey responses and measured EHR access data were adjusted for these sociodemographic characteristics that could potentially associate with a tendency towards accessing the EHR to monitor their health or share health information with others, and for baseline CRS and GRS. We assessed whether information exchange behaviors differed among study participants, by GRS disclosure, or by high versus low GRS (‘Expansion of Results’ in Appendix C), using odds ratio (OR) with 95% confidence interval (CI) (for nominal logistical regression analyses), or mean or beta with standard error (SE) (for continuous variables or ordinal logistic regression analyses, respectively). A two-sided *P*-value of <0.05 was used to determine statistical significance in this exploratory secondary analysis. Post-hoc power analyses and multivariate analyses were also performed (‘Post-hoc power analyses’ and ‘Multivariate analyses’ in Appendix B).

## 3 Results

Participants in both study groups had similar sociodemographic characteristics (Table 1); the majority of participants (~60%) reported some college education or higher, similar to national reports on distribution of college education in survey respondents (Ryan and Bauman, 2016). All data were adjusted for these baseline sociodemographic characteristics, using multivariate logistic regression.

**Table 1:** Sociodemographic characteristics of study participants (n = 203), Olmsted County, MN; 2013–2015.

	CRS n = 100	GRS n = 103
Age (years)	59.4 ± 5.3	59.4 ± 4.9
Female sex, n (%)	51 (51.0%)	55 (53.4%)
Family history of CHD, n (%)	30 (30.0%)	25 (24.3%)
Some college education or higher, n (%)	67 (67.0%)	58 (56.3%)
GRS	1.11 ± 0.30	1.14 ± 0.29
CRS, 10-year probability (%)	8.48 ± 3.76	8.56 ± 4.47

CHD = coronary heart disease; CRS = conventional risk score (Framingham risk score); GRS = genetic risk score.

### 3.1 Risk recall

One third of study participants correctly recalled their disclosed CHD risk, with no significant difference between the CRS and GRS groups (Appendix Fig. A.2).

### 3.2 Identifying EHR users for CHD risk

Approximately 70% of study participants accessed their EHR overall (see ‘Evolution of EHR Use in the MI-GENES Study’ in Appendix D). Individuals who reported accessing the patient portal to review their CHD risk information at three or six months after risk disclosure (in response to question 5 in Appendix Table A.1) were labelled as “EHR Users for CHD Risk” (EUCRs). Individuals who self-reported NOT logging into the patient portal to review their CHD risk information were labelled as “NOT EHR Users for CHD Risk” (NEUCRs). There was no difference in risk recall between EUCRs and NEUCRs (data not shown). Self-reported EHR access associated with objectively quantified frequency of EHR access (Table 2) (‘Expansion of Results’ in Appendix C). In particular, among both GRS participants (71(10) versus 34(7); *P* = 0.0029) and CRS participants (69(12) versus 29(6); *P* = 0.0063), EUCRs had higher quantified EHR access frequencies than NEUCRs (Table 3). This suggested that EUCRs may access the EHR to review their CHD risk information frequently.

**Table 2:** Association of objectively quantified frequency of EHR access with responses to information exchange survey questions; Olmsted County, MN; 2013–2015.

	Total frequency of EHR access via patient portal login based on							
	Yes/no response to each individual information exchange survey question							
	3 months after risk disclosure				6 months after risk disclosure			
	n^a^	Yes	No		n	Yes	No	
	(Yes)	Mean (SE)	Mean (SE)	*P*-value	(Yes)	Mean (SE)	Mean (SE)	*P*-value
Survey questions regarding patient portal access								
1. Used e-mail or the Internet to communicate with a doctor or doctor's office?	93	63 (7)	27 (6)	<0.0001^b^	88	68 (6)	23 (6)	<0.0001^b^
2. Kept track of personal health information such as care received, test results, or upcoming medical appointments?	125	59 (5)	16 (7)	<0.0001^b^	119	56 (6)	23 (7)	<0.0001^b^
3. Do you have access to your Mayo Clinic Patient Portal?^c^	162	53 (5)	8 (9)	<0.0001^b^	148	56 (5)	9 (8)	<0.0001^b^
4. If yes, did you sign-up for the Patient Portal after enrollment in this study?	9	37 (20)	46 (5)	0.6548	16	37 (15)	43 (5)	0.6292
5. Did you use the Patient Portal to access information related to your risk of having a heart attack as a part of this study?	32	70 (10)	36 (5)	0.0023^b^	35	70 (10)	37 (5)	0.0025^b^

Survey questions regarding internet use								
1. Have you looked for any information about how your personal health habits, such as your diet and exercise, affect your chances of getting a heart attack?	120	47 (6)	34 (7)	0.1297^b^	84	44 (7)	41 (6)	0.7045^b^
2. Have you looked for any information about how genetic factors affect your chances of getting a heart attack?	71	52 (7)	36 (6)	0.0772^b^	42	51 (9)	40 (5)	0.2683^b^
3. In the past 3 months, have you used the Internet to look for information about heart disease for yourself?^d^	34	64 (10)	38 (5)	0.0178^b^	29	39 (11)	43 (5)	0.7052
4. Is there a specific internet site you like to go to for information about heart disease?^d^	25	59 (12)	42 (5)	0.1797^b^	15	38 (16)	43 (5)	0.7633
5. Looked for health or medical information?	132	52 (6)	26 (7)	0.0022^b^	132	52 (6)	26 (7)	0.0031^b^
6. Used a website to help you with your diet, weight, or physical activity?	80	51 (7)	38 (6)	0.1276^b^	64	49 (8)	40 (6)	0.3081^b^
7. Do anything else health-related on the Internet?	65	45 (8)	41 (6)	0.6301^b^	69	54 (7)	36 (6)	0.0453^b^
8. Visit an Internet Supplemental site to learn specifically about heart disease?^d^	30	54 (11)	40 (5)	0.2417^b^	18	44 (14)	42 (5)	0.8794

Survey questions regarding information sharing								
1. Have you discussed your risk of having a heart attack with others?	170	42 (5)	45 (11)	0.7858	169	42 (5)	44 (11)	0.9089
2. Who did you talk to about your results?	Not convertible to ‘Yes’/‘No’ question.							
3. Did you share your risk of having a heart attack with your parents?	36	44 (10)	43 (5)	0.8887	42	38 (10)	44 (5)	0.6160
4. Did you share your risk of having a heart attack with your siblings?	83	35 (7)	48 (6)	0.1334	91	40 (6)	44 (6)	0.6449^b^
5. Did you share you risk of having a heart attack with your spouse?	155	42 (5)	43 (9)	0.9245	153	43 (5)	42 (9)	0.8.302
6. Did you share you risk of having a heart attack with your children?	101	37 (6)	49 (7)	0.1706	98	43 (6)	42 (7)	0.9436
7. Did you share your CHD risk with your extended family?	20	55 (13)	41 (5)	0.3079	21	51 (13)	41 (5)	0.4686^b^
8. Did you share or intend to discuss your CHD risk with your PCP?	140	46 (6)	34 (8)	0.1906^b^	135	41 (6)	46 (8)	0.5485^b^
9. Did you use Facebook to share your CHD risk?	0	–	42 (5)	–	2	22 (42)	43 (5)	0.6233
10. Did you use Twitter to share your CHD risk?	0	–	42 (5)	–	0	–	42 (5)	–
11. Did you use other social networking services to share your risk of having a heart attack with others?	1	91 (59)	42 (5)	0.4043	2	Unstable	Unstable	Unstable
12. Have you encouraged others to be screened for risk of having a heart attack?	108	42 (6)	43 (7)	0.8647	108	51 (6)	32 (7)	0.0269^b^

Survey questions regarding social network								
1. Do you have friends or family members that you talk to about your health?	172	41 (5)	47 (11)	0.6514^b^	163	46 (5)	24 (10)	0.0381^b^
2. Do any community organization(s) provide you with information on health?	44	32 (9)	45 (5)	0.1991^b^	42	59 (9)	38 (5)	0.0368^b^

The table reports the mean ± standard error for the objectively quantified total patient portal login frequency during the entire study for two columns: ‘Yes’ and ‘No’. Each column represents the ‘Yes’ or ‘No’ responses to individually listed numbered information seeking and sharing survey questions.

EHR = Electronic Health Record; PCP = primary care provider; SE = Standard Error.

a Number of study participants who responded ‘Yes’ to the corresponding survey question (total number of study participants = 203).

b Power 80% or greater.

c For individuals who reported not having have access to their patient portal, the mean number of visits to this portal was 8 at three months or 9 at six months after risk disclosure (see ‘Limitations of Assessing EHR Access’ in Appendix D).

d Question 8 differs from question 3 by investigating whether the trial participant intentionally sought out internet websites to specifically learn about heart disease, whereas question 3 can capture passive internet use for heart disease information, which can occur while browsing webpages for other reasons; question 8 differs from question 4, as the latter investigates the consistent use of a particular internet website to learn about heart disease.

**Table 3:** **Quantified patient portal access frequency for subcategories of CRS and GRS participants;** Olmsted County, MN; 2013-2015

Participants	Mean	Standard error	P-value
CRS	38	6	0.3099 ^a^
GRS	47	6	
CRS NEUCRs	30	7	0.0063 ^***a***^
CRS EUCRs	70	13	
GRS EUCRs	71	10	0.0029 ^***a***^
GRS NEUCRs	35	7	

The table reports the mean and standard error for participants’ quantified frequency of patient portal access. ^***a***^ Power 80% or greater. CRS: conventional risk score; EUCRs: EHR Users for CHD Risk; GRS: genetic risk score; NEUCRs: NOT EHR Users for CHD Risk.

### 3.3 EHR access associated with other information exchange behaviors

**Fig. 2:**
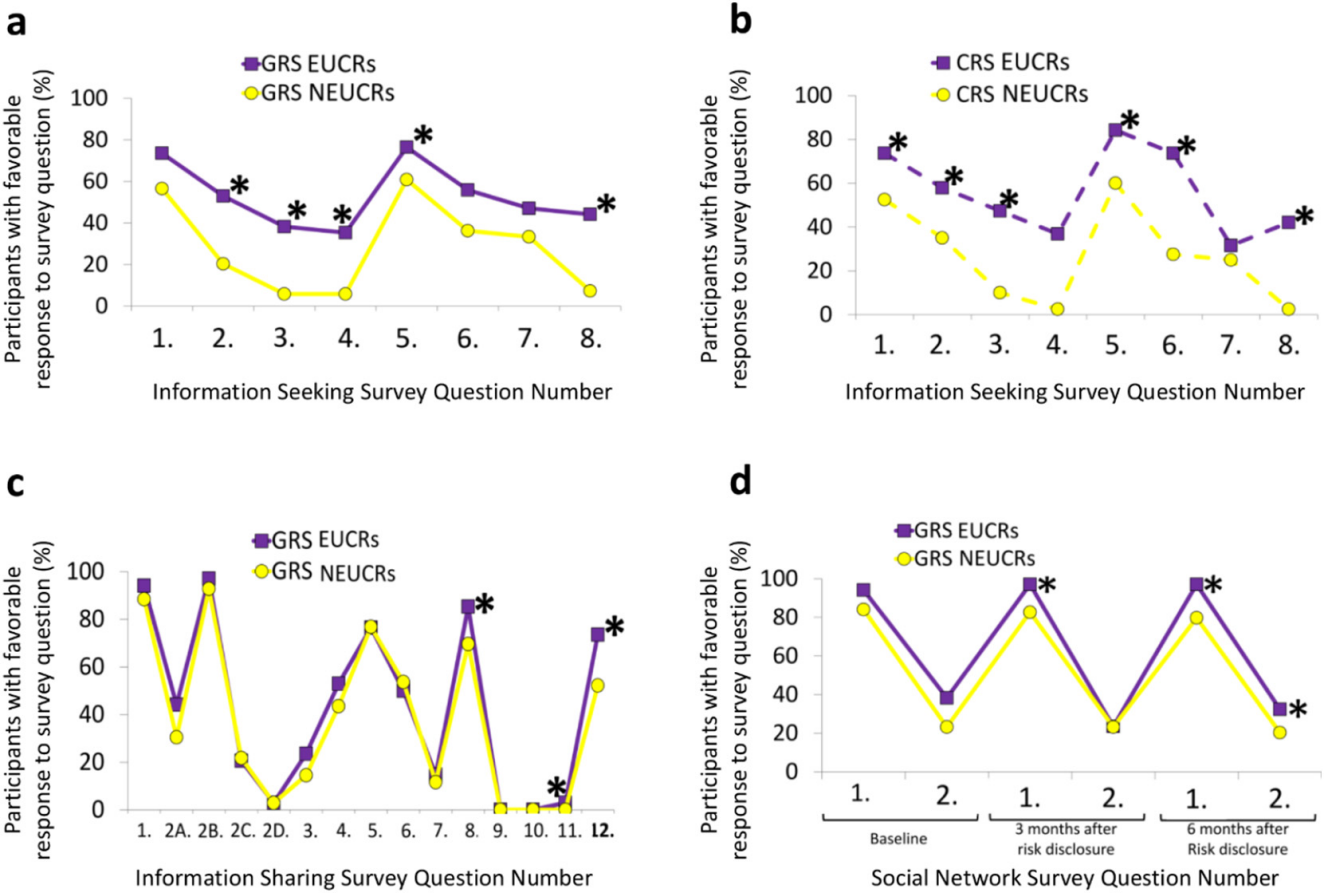
Information Exchange Trends in Social Networks (Olmsted County, MN; 2013–2015). Internet use for information seeking outside of the patient portal at 3 months after risk disclosure for (a) GRS participants, and (b) CRS participants. (c) Information sharing at 3 months after risk disclosure for GRS participants. (d) Social network at baseline, 3 months, and 6 months after risk disclosure for GRS participants. CRS = conventional risk score; EUCRs = EHR Users for CHD Risk; GRS = genetic risk score; NEUCRs = NOT EHR Users for CHD Risk. * *P* < 0.05.

**Fig. 3:**
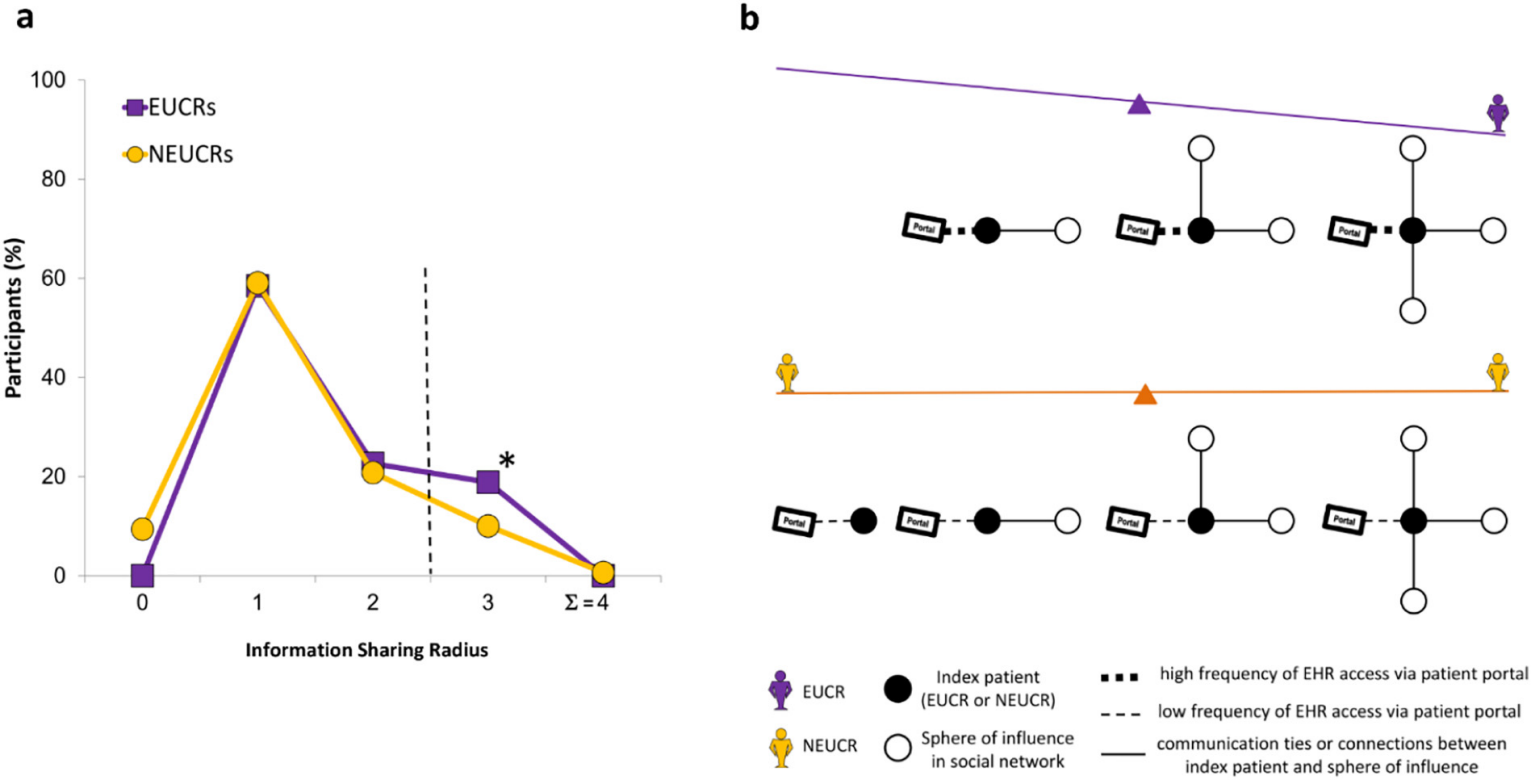
A Provisional Information Sharing Radius Score and a Conceptual Model for Information Exchange in Social Networks (Olmsted County, MN; 2013–2015). (a) Percentage of participants with a particular information sharing radius: EUCRs as a single class among all 203 trial participants had a wider information sharing radius than NEUCRs, with a maximum possible score of 4 (∑; sum) demonstrated to the right of the vertical dashed line. * *P* < 0.05. (b) A conceptual model of EUCRs and NEUCRs in social networks: EUCRs had a high frequency of quantified EHR access via a patient portal, with a skew towards a higher number of communication ties or connections; NEUCRs had a low frequency of quantified EHR access via a patient portal, with no skew towards a higher number of ties. EUCRs: EHR Users for CHD Risk; NEUCRs: NOT EHR Users for CHD Risk.

Self-reported EHR access associated with other measures of information exchange behaviors. Within both randomized groups, EUCRs were more likely than NEUCRs to report seeking health information online outside of the EHR (information seeking) (Baseline: Appendix Fig. A.3; 3 months post-disclosure: Fig. 2a–b; 6 months post-disclosure: Appendix Fig. A.4a–b) and sharing health information in their social networks (Fig. 2c–d, Appendix Fig. A.4c–d, and Appendix Fig. A.5) (‘Expansion of Results’ in Appendix C). This was noted at baseline and was observed throughout the study. This suggested a general tendency towards information exchange in EUCRs in both randomized groups even prior to risk disclosure, which increased following CHD risk disclosure. EUCRs as a single class among all 203 trial participants were more likely than NEUCRs to encourage others to be screened for their CHD risk (OR 2.936, CI 1.443–5.973, *P* = 0.0030) (data not shown), and tended to have a wider information sharing radius than NEUCRs three months after CHD risk disclosure (beta 0.308 ± 0.157, *P* = 0.0498) (Fig. 3a), with a similar trend at six months post-disclosure (beta 0.267 ± 0.156, *P* = 0.0870). Quantified EHR access frequency also associated with self-reported internet use outside of the EHR and information sharing via interpersonal communication in social networks (Table 2). Thus, both self-reports of EHR access to review CHD risk information and quantified frequent EHR access associated with sharing of CHD risk information in social networks.

### 3.4 Conceptual model

We developed a conceptual model of EUCRs and NEUCRs in social networks (Fig. 3b). Based on the frequency of EHR access (Tables 3 and 4), EUCRs had wider connecting lines to the patient portal, representing a higher frequency of EHR access or a stronger information seeking relationship of EUCRs with the EHR. In contrast, NEUCRs had narrow connecting lines to the EHR, representing a lower frequency of EHR access or a weaker information seeking relationship with the EHR. Upon inspection of the data from the information sharing radius (Fig. 3a), we discovered that EUCRs reported forming at least 1 communication tie (i.e., a sharing radius of 1) and tended to report a greater number of communication ties (a skew towards 3 communication ties or a sharing radius of 3 reported by one in five EUCRs). On the other hand, one in ten NEUCRs reported forming 0 communication ties (i.e., a sharing radius of 0); one in ten NEUCRs also reported forming 3 communication ties, with no skew in the data.

### 3.5 Impact of GRS

There was no difference between CRS participants and GRS participants (37(6) versus 46(6); *P* = 0.2834) in the actual quantified EHR access frequency throughout the study (Table 3). Similarly, there was no difference between EUCRs in the CRS group (n = 19, 19%) and EUCRs in the GRS group (n = 34, 33%) (70(13) versus 71(10); *P* = 0.9339) in the actual quantified EHR access frequency throughout the study (Table 3). However, GRS participants were more likely than CRS participants to log into the patient portal (Appendix Table A.5), and to report accessing the EHR to specifically seek their CHD risk (OR 2.99; CI: 1.35,7.04; *P* = 0.0171) (Appendix Fig. A.6a) at 6 months post-disclosure. Notably, EUCRs in the GRS group were even more likely than EUCRs in the CRS group to seek health information outside of the EHR and share health information in social networks, e.g., with friends or family (OR 15.74; CI: 1.54,160.97; *P* = 0.0201), supporting the impact of GRS disclosure (Appendix Fig. A.6b) (‘Expansion of results’ in Appendix C).

## 4 Discussion

EHR access by individuals may play a role in health promotion in their non-digital social and health networks. In this secondary analysis of the MI-GENES trial, we hypothesized that individuals who self-reported EHR access to seek their CHD risk information actually accessed the EHR frequently and shared the gleaned health information widely in their social networks. Those who self-reported EHR access to seek their CHD risk information (EUCRs) more frequently accessed the EHR, sought health information online outside of the EHR, shared health information in their social networks, encouraged others to be screened for their CHD risk, and had a wider information sharing radius than individuals who did not log in to review their risk (NEUCRs). Our conceptual model illustrated the potential role of EUCRs in dissemination of health information in social networks.

Survey responses were key to understanding reasons for accessing the EHR. For example, two thirds of participants did not correctly recall their disclosed CHD risk. Persistent poor recall may have led to repeated EHR access to retrieve CHD risk information. However, there was no difference in risk recall between EUCRs and NEUCRs, suggesting that poor recall alone does not explain the behavior of EUCRs. The EUCRs accessed their EHR frequently (based on objective quantification) and reported other information exchange behaviors; several individuals accessed their EHR to seek their CHD risk information only after receiving their genetic risk information. With each login, there was potential to again retrieve risk information; perhaps each review episode corresponded with further sharing of the CHD risk with others in the study participants' social network. Notably, information seeking and sharing have both been perceived as health-promotive activities by mediating bidirectional social information acquisition (van den Putte et al., 2011; Lambert and Loiselle, 2007; Stoffel et al., 2008). Regardless of the source of motivation for sharing their risk information, EUCRs overall disseminated genetic risk information more often than NEUCRs to multiple spheres of influence - among their family, friends, co-workers, and other members of their social and health networks, including primary care providers. An ‘information sharing radius’ may serve as a provisional score to estimate the extent of such health information sharing by EUCRs in their networks. There is potential for a ripple effect in a ‘cascade of influence’ (Kempe et al., 2015), in which information shared with target individuals is disseminated to others in their spheres of influence without ever having direct contact with the original source of the information.

Importantly, studies have revealed that a GRS can lead to reclassification of CHD risk estimates (Ganna et al., 2013; Tikkanen et al., 2013; Hughes et al., 2012; Ripatti et al., 2010; Iribarren et al., 2016; Khera et al., 2016), and is associated with future CHD events and clinical response to statin therapy (Mega et al., 2015), and initiation of new medications or other behavior modification (Kullo et al., 2016; Christensen et al., 2016; Scheinfeldt et al., 2016). Further, other studies on genetic risk disclosure for conditions such as familial hypercholesterolemia, other cardiovascular disorders, or hereditary cancers suggest that patients who receive their genetic risk information often share this information with family members and this may encourage preventive screening and to raise awareness (Christophe et al., 2009; Hallowell et al., 2011; Smart, 2010; Sturm, 2016) (‘Genetic versus other health information’ in Appendix E). The impact of GRS disclosure for CHD in the MI-GENES study indeed bears similarities with other studies of information exchange by patients after disclosure of genetic risk information, e.g., sharing cancer genetic risk information with family members, or sharing diabetes or Alzheimer's genetic risk information with family members, friends, co-workers, and other health care providers (Christophe et al., 2009; Hallowell et al., 2011; Mills et al., 2015; van Esch et al., 2012a; van Esch et al., 2012b; Whitford et al., 2009; Ashida et al., 2009; Patenaude et al., 2006). Similarities in sharing patterns for conditions considered non-modifiable (e.g., some hereditary cancers or Huntington's disease) and those considered modifiable (e.g., CHD or diabetes) may be due to expectations based on shared familial risk (which could propagate genetic risk screening among familial networks), and differences may be due to expectations based on collaborative lifestyle habits as well as sources of tangible, emotional, and social support.

We developed a conceptual model of the role of EUCRs and NEUCRs in social networks (Fig. 3b) (‘Conceptual Model’ in Appendix F). Our model development was different from that of Zhu et al. (2016), in which EHR access was used to identify individual patients' professional health care teams as social networks based on access of the EHR by the patient's health care providers. Our model suggests that assessment of self-reported EHR access to obtain one's CHD risk information could identify individuals (EUCRs; Fig. 3b, purple schematic) who may form greater numbers of communication ties in their social networks than observed for NEUCRs. Further, the model visually captures our results that indicate a relationship between high frequency of quantified EHR access (or a strong information seeking relationship with the EHR) by EUCRs and a skew towards higher numbers of communication ties with individuals in their social networks.

Overall, our findings suggest that individuals labelled as EUCRs may help disseminate health information in (and thereby influence) their social networks. Such individuals could be termed ‘central nodes or hubs’ in formal social network analyses. In social networks, individuals are considered as nodes and interpersonal communication and relationships as connections between nodes (Aral and Walker, 2012; Borgatti et al., 2009; Chen et al., 2013; Zhang et al., 2016). Central nodes or hubs, also described as influencers, disseminators, or spreaders, have extensive connections in their social networks, exhibiting high levels of information exchange, and are thought to be well-positioned to move information around and influence the social network (Aral and Walker, 2012; Borgatti et al., 2009; Chen et al., 2013; Zhang et al., 2016). Interpersonal communication among individuals in social networks may therefore be effective for health promotion and outcomes (van den Putte et al., 2011; Christophe et al., 2009; Christakis and Fowler, 2008; Brunson, 2013; Social Networks and Vaccination Decisions, 2007; Sun, 2014; Koehly et al., 2009). Formal social network analyses could be performed to further analyze the potential role of EUCRs as central nodes in their social networks.

Study limitations include a relatively small (n = 203) and relatively well-educated sample. All participants were whites – the primary population in which the GRS for CHD was developed. Survey responses could reflect an element of recall bias. Nevertheless, this secondary analysis of the MI-GENES study is the first to report that frequency of EHR access can be used to identify individuals who are more likely to share CHD risk information in their social networks and thereby help promote CHD prevention in population health.

## Appendix A Supplementary data

Supplementary material Image 1
