# Supplementary material for: Electronic health record access by patients as an indicator of information seeking and sharing for cardiovascular health promotion in social networks: Secondary analysis of a randomized clinical trial
Source: Prev Med Rep. 2019 Jan 14;13:306–13. doi: 10.1016/j.pmedr.2018.12.011 (PMC6365362; doi:10.1016/j.pmedr.2018.12.011)
Supplement: Supplementary file 1 — Supplementary material [file mmc1.pdf]

APPENDIX

Electronic Health Record Access by Patients as an Indicator of Information Seeking and Sharing for Cardiovascular Health Promotion In Social Networks: Secondary Analysis of a Randomized Clinical Trial

Running title: EHR Access For Health Promotion

Sherry-Ann N. Brown, MD, PhD, Hayan Jouni, MD, Iftikhar J. Kullo, MD*

Department of Cardiovascular Diseases, Mayo Clinic, Rochester, MN

***Corresponding author:** Iftikhar J. Kullo, M.D., Department of Cardiovascular Diseases, Mayo Clinic, 200 First Street SW, Rochester, MN 55905, FAX: (507) 266-1617; TaEL: (507) 266-3964, E-mail:Kullo.Iftikhar@mayo.edu.

APPENDIX A Tables and Figures

Appendix Table A.1 Survey questions related to EHR access via a Patient Portal

	Yes	No
The following pertains to questions 1 and 2. In the last 3 months, have you used the Internet for any of the following reasons?		
1. Used e-mail or the Internet to communicate with a doctor or doctor's office?	☐	☐
2. Kept track of personal health information such as care received, test results, medical appointments?	☐	☐
3. Do you have access to your Patient Portal?	☐	☐
4. If yes, did you sign-up for the Patient Portal after enrollment in this study?	☐	☐
5. Did you use the Patient Portal to access information related to your risk of having a heart attack as a part of this study?	☐	☐

EHR = electronic health record.

Appendix Table A.2 Survey questions related to internet use outside of the patient portal

	Yes	No
1. Have you looked for any information about how your personal health habits, such as your diet and exercise, affect your chances of getting a heart attack?	☐	☐
2. Have you looked for any information about how genetic factors affect your chances of getting a heart attack?	☐	☐
3. In the past 3 months, have you used the Internet to look for information about heart disease for yourself? [a]	☐	☐
4. Is there a specific internet site you like to go to for information about heart disease? [a]	☐	☐
In the last 3 months, have you used the Internet for any of the following reasons?	☐	☐
5. Looked for health or medical information?	☐	☐
6. Used a website to help you with your diet, weight, or physical activity?	☐	☐
7. Do anything else health-related on the Internet?	☐	☐
8. Visit an Internet Supplemental site to learn specifically about heart disease? [a]	☐	☐

[a] Question 8 differs from question 3 by investigating whether the trial participant intentionally sought out internet websites to specifically learn about heart disease, whereas question 3 can capture passive internet use for heart disease information, which can occur while browsing webpages for other reasons. Question 8 differs from question 4, as the latter investigates the consistent use of a particular internet website to learn about heart disease.

Appendix Table A.3 Survey questions related to information sharing in social networks

**Social Network**	Yes	No
1. Do you have friends or family members that you talk to about your health?	☐	☐
2. Do any community organization(s) provide you with information on health?	☐	☐

**Information Sharing**	Not at all	Very few	Some	A fair number	Frequently
1. Have you discussed your risk of having a heart attack with others?[a]	☐	☐	☐	☐	☐
2. Who did you talk to about your results? ☐ A. Friends ☐ B. Family members ☐ C. Co-workers ☐ D. Other					

	Yes	No
3. Did you share your risk of having a heart attack with your parents?	☐	☐
4. Did you share your risk of having a heart attack with your siblings?	☐	☐
5. Did you share you risk of having a heart attack with your spouse?	☐	☐
6. Did you share you risk of having a heart attack with your children?	☐	☐
7. Did you share your CHD risk with your extended family?	☐	☐
8. Did you share or intend to discuss your CHD risk with your PCP?	☐	☐
9. Did you use Facebook to share your CHD risk?	☐	☐
10. Did you use Twitter to share your CHD risk?	☐	☐
11. Did you use other social networking services to share your risk of having a heart attack with others? [b]	☐	☐

	Not at all	Very few	Some	A fair number	Frequently
12. Have you encouraged others to be screened for their CHD risk? [a]	☐	☐	☐	☐	☐

CHD = coronary heart disease, PCP = Primary care provider. [a] Due to a low frequency of “unfavorable” responses for “Not at all” and “Very few”, a binary scale was used: a score of 1 was given for “unfavorable” responses, and 2 for “favorable responses” (i.e., “Some”, “A fair number”, “Frequently”, and “Yes”). [b] A question about sharing CHD risk on social networks online was also added to assess whether information sharing spread beyond non-digital social networks into digital social networks.

Appendix Table A.4 System for analysis of quantified EHR login via the patient portal

Favorable survey question response	Total measured EHR Login via patient portal	
Yes	1<br>0<br>100<br>20<br>69	mean ± standard error
No	0<br>2<br>0<br>50<br>18	mean ± standard error

EHR = Electronic health record

Appendix Table A.5 Impact of GRS disclosure on quantified EHR access; Olmsted County, MN; 2013-2015

EHR access	CRS, n (%)	GRS, n (%)	All participants, n(%)	OR	CI	P-value
0	19 (19)	11 (11)	30 (15)	0.40	0.17-0.92	0.0351
1-50	61 (62)	62 (60)	123 (61)	0.98	0.54-1.78	0.9488
51-100	11 (11)	19 (18)	30 (15)	1.87	0.83-4.40	0.1384
>100	8 (8)	11 (11)	19 (9)	1.51	0.57-4.18	0.4114

CI = confidence interval, CRS = conventional risk score, EHR = electronic health record, GRS = genetic risk score, n = number of participants, OR = odds ratio.

Appendix Table A.6 Multivariate logistic regression with sociodemographic characteristics as potential predictors

	Quantified EHR Login (OR)[a]		
	0	51-100	100-200
Age (years)	10.01	0.98	0.940
Female sex, n (%)	10.08	2.79[b]	20.100
Family history of CHD, n (%)	1.26	0.90	3.790[b]
College education or higher, n (%)	0.33[b]	1.41	1.240
GRS	0.26	1.74	1.860
CRS, 10-year probability	0.95	0.87	10.020

[a] Multivariate logistic regression with odds ratio (OR) indicated no potential predictors for electronic health record (EHR) login frequency of 1-50 counts for TotalLogin over the course of the trial. [b] $p<0.05$. CHD: coronary heart disease; CRS: conventional risk score (Framingham risk score); GRS: genetic risk score.

Appendix Table A.7 Correlations among total frequencies of Patient Portal login

Timeframe	by Timeframe	Correlation	Lower 95%	Upper 95%
Total Login during trial	Total V1toV2	0.651	0.563	0.724
Total Login during trial	Total V2toV3	0.713	0.637	0.775
Total Login during trial	Total V3to V4	0.773	0.711	0.823
Total Login during trial	Total V4toPostV4	0.952	0.927	0.968
Total Login V1toV2	Total Login V2toV3	0.604	0.508	0.685
Total Login V1toV2	Total Login V3to V4	0.512	0.402	0.607
Total Login V1toV2	Total Login V4toPostV4	0.435	0.316	0.541
Total Login V2toV3	Total Login V3to V4	0.612	0.523	0.696
Total Login V2toV3	Total Login V4toPostV4	0.736	0.623	0.819
Total Login V3to V4	Total Login V4toPostV4	0.729	0.614	0.814

Total Login during trial = total frequency of Patient Portal login for the duration of the entire trial. Total Login V1toV2 = Total Login frequency of Patient Portal login from baseline to initial risk disclosure. Total Login V2toV3 = Total Login frequency of Patient Portal login from initial risk disclosure to three months after initial risk disclosure. Total Login V3toV4 = Total Login frequency of Patient Portal login from three to six months after initial risk disclosure. Total Login V4toPostV4 = Total Login frequency of Patient Portal login from delayed risk disclosure of GRS to CRS participants to three months after delayed risk disclosure.

Appendix Figure A.1 Distribution of GRS among study participants, with minimum 0.50 and minimum 2.01. GRS = genetic risk score.

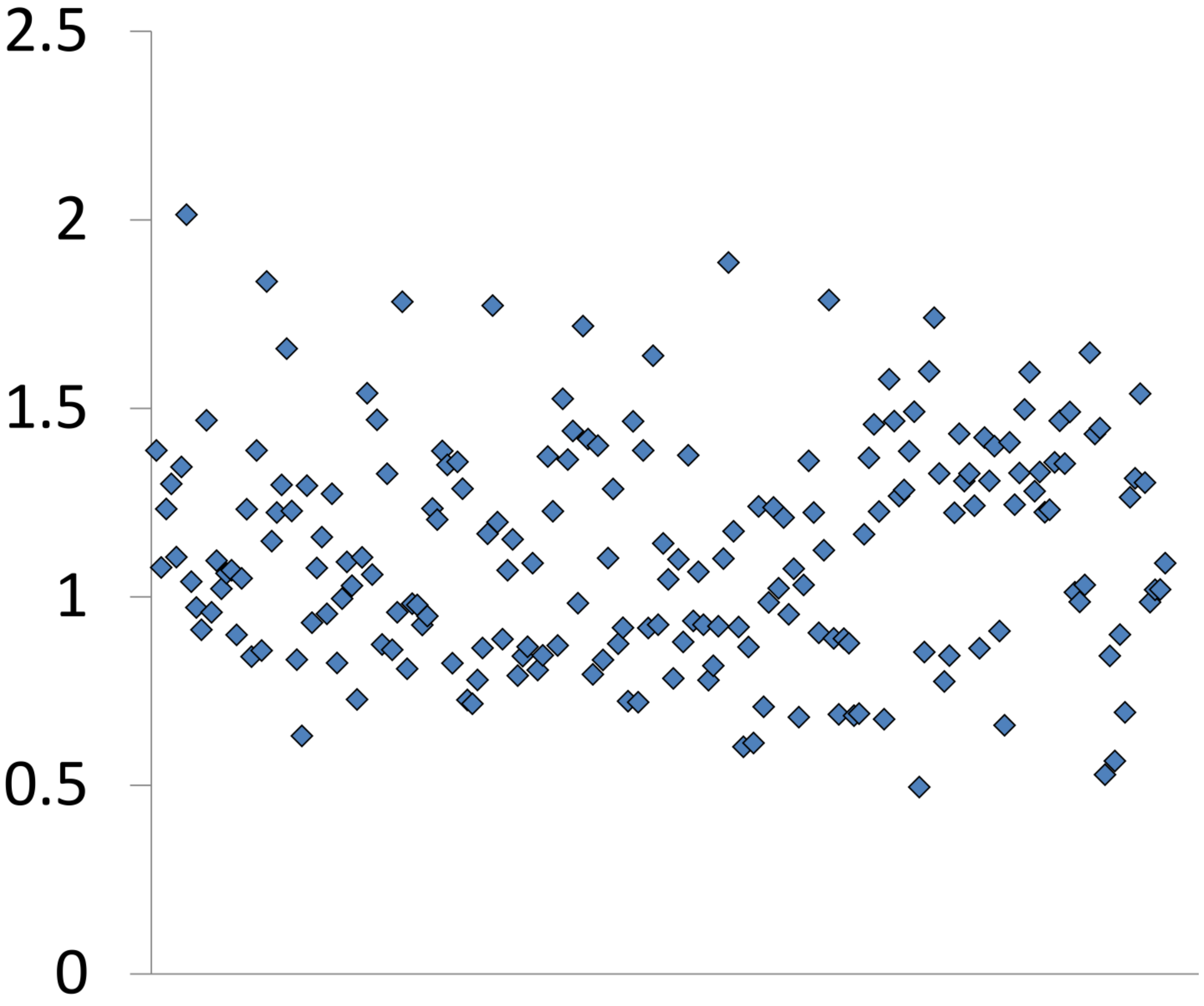

Appendix Figure A.2 Precise risk recall in study participants, with no difference (P=NS) between the CRS (34%) and GRS (33%) groups. CRS = conventional risk score; GRS = genetic risk score; NS = no significance.

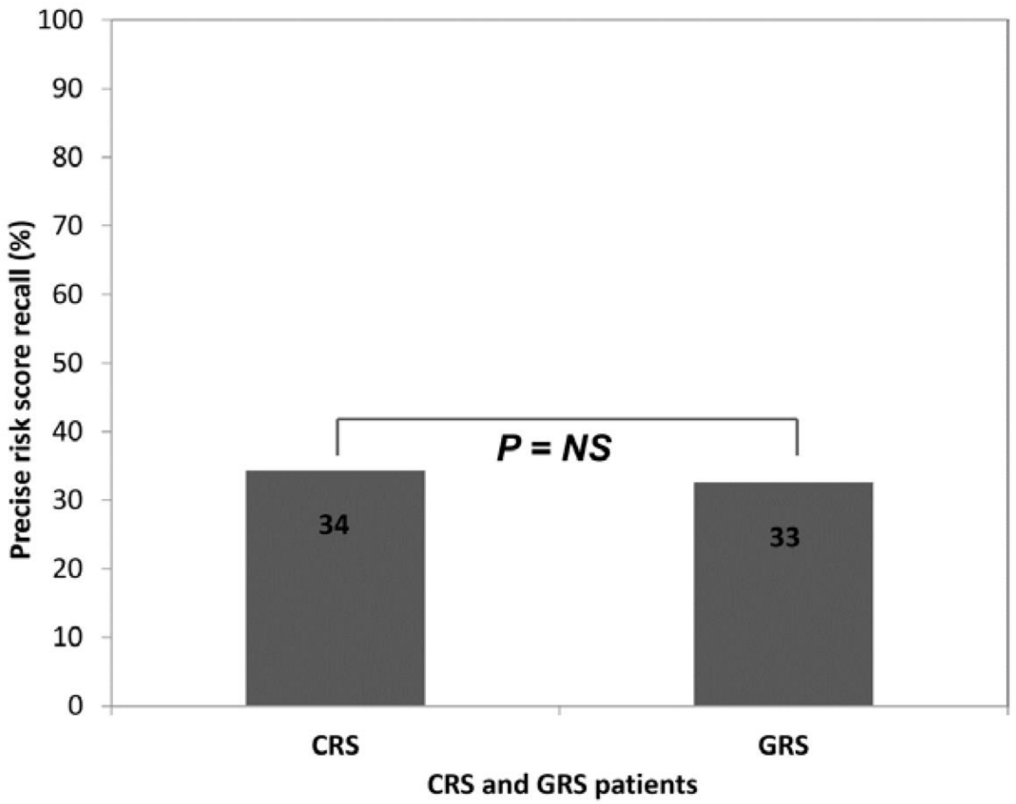

Appendix Figure A.3 Baseline internet use. Internet use prior to risk disclosure for participants in the (a) GRS group (these questions were not adequately powered ≥80%), and (b) the CRS group (these questions were not adequately powered ≥80%). CRS = conventional risk score; EUCRs = EHR Users for CHD Risk; GRS = genetic risk score; NEUCRs = NOT EHR Users for CHD Risk. * *P* <0.05.

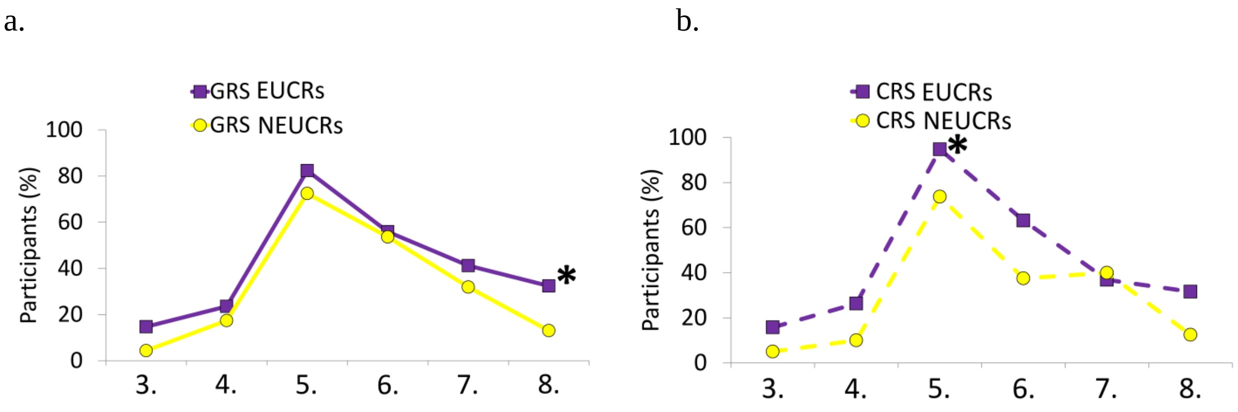

Appendix Figure A.4 Information seeking and sharing at 6 months after risk disclosure. Information seeking at 6 months after risk disclosure for (a) GRS participants (questions 2, 4, and 8 were adequately powered ≥80%), and (b) CRS participants (question 1 was adequately powered ≥80. Information sharing at 6 months after risk disclosure for (c) GRS participants (no questions were adequately powered ≥80%), and (d) CRS participants (no questions were adequately powered ≥80%). CRS = conventional risk score; EUCRs = EHR Users for CHD Risk; GRS = genetic risk score; NEUCRs = NOT EHR Users for CHD Risk. * P <0.05.

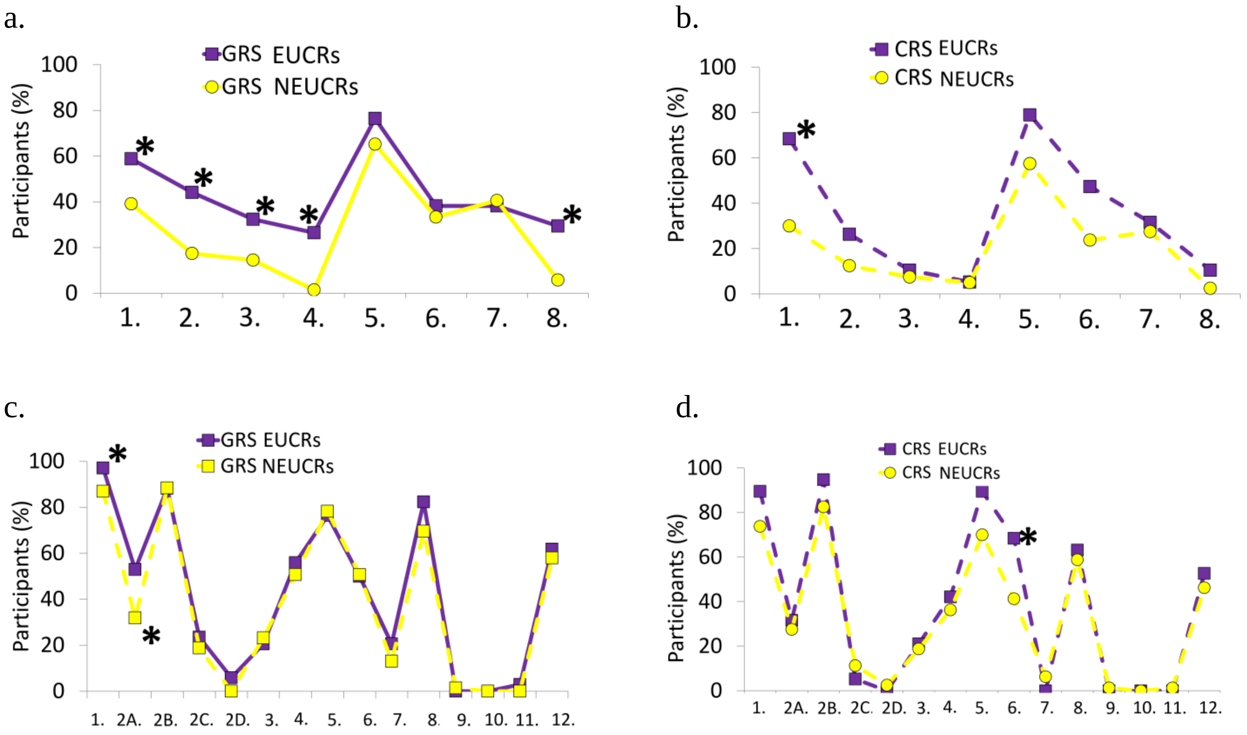

Appendix Figure A.5 Information Exchange Trends in Social Networks. (a) Information sharing at 3 months after risk disclosure for CRS participants (no questions were adequately powered ≥80%); (b) Social network at baseline, 3 months, and 6 months after risk disclosure CRS participants (no questions were adequately powered ≥80%). Various numbered survey questions (see Appendix Table A.2 for Information seeking; and Appendix Table A.3 for Information Sharing and Social network) are represented on the x-axes, with percentage of study participants on the y-axes. EUCRs were those individuals who responded ‘yes’ to accessing the patient portal to look for their CHD risk information during the study (Olmsted County, MN; 2013-2015); NEUCRs responded ‘no’. CRS = conventional risk score; GRS = genetic risk score. * *P* <0.05.

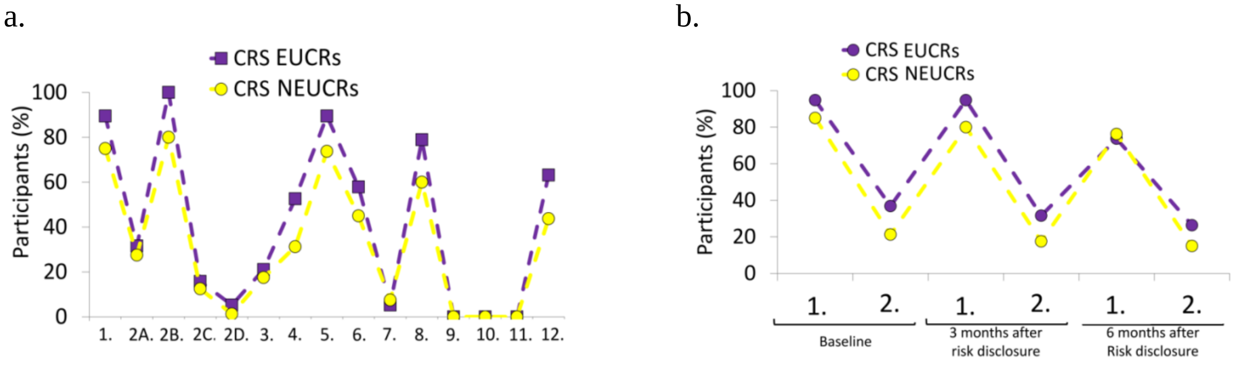

Appendix Figure A.6 Impact of GRS disclosure**.** (a) EHR access by all study participants in the GRS group and all study participants in the CRS group to obtain CHD risk information at three and six months after risk disclosure (this question was adequately powered ≥80% at six months after risk disclosure but not at 3 months post-disclosure)(question 5 in Appendix Table A.1). (b) Information exchange in EUCRs at 6 months after risk disclosure (these questions were underpowered <80%); various numbered survey questions (from Appendix Table A.2 for Information seeking and Appendix Table A.3 for Social network) are represented on the x-axes, with percentage of study participants on the y-axes. EUCRs were those individuals who responded 'yes' to accessing the patient portal to look for their CHD risk information during the study (Olmsted County, MN; 2013-2015); NEUCRs responded 'no'. CRS = conventional risk score; GRS = genetic risk score. * P <0.05.

a.

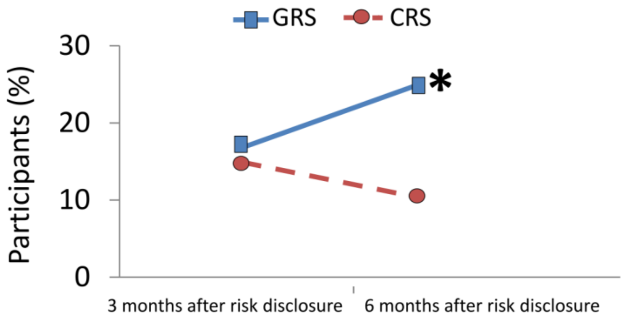

b.

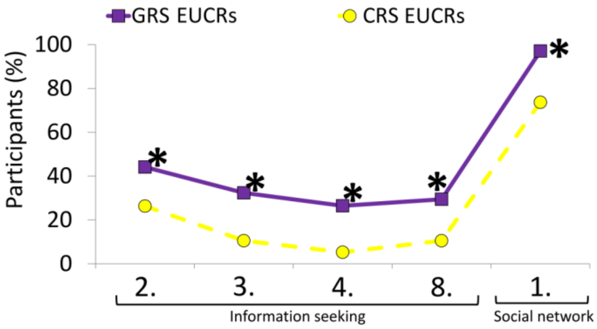

APPENDIX B

Post-hoc power analyses

Post-hoc power analyses were performed for all survey questions to determine retrospectively whether the study was adequately powered for each question (≥80%; using an online application at http://clincalc.com/stats/Power.aspx). The obtained sample size and observed effect size for the CRS and GRS groups were used to determine power for an alpha of 0.05, with the assumption that the sample effect size for each survey question was equivalent to that of the population.

The study was overall adequately powered (≥80%) (Tables 2-4). Analyses revealed adequate power (≥80%) for the relationship between quantified EHR access and subjective reports of information exchange at three and six months post-disclosure (Table 2), and for comparisons of quantified EHR access within and between the CRS and GRS groups, with one exception (Appendix Table A.6). Given the small number of "EHR users for CHD risk" (EUCRs) in each group, the comparisons between EUCRs in the GRS group and EUCRs in the CRS group were underpowered (Appendix Table A.6). For GRS participants' internet use for information seeking outside of the patient portal at 3 months after risk disclosure, questions 2, 3, 4, and 8 were adequately powered ≥80%. For CRS participants' internet use for information seeking outside of the patient portal at 3 months after risk disclosure, questions 2, 3, 4, 6, and 8 were adequately powered ≥80. For GRS participants' information sharing at 3 months after risk disclosure for GRS participants, no questions were adequately powered ≥80%. For GRS participants' social network at baseline, 3 months, and 6 months after risk disclosure, no questions were adequately powered ≥80%.

Comparing subjective reports of internet use between EUCRs and “NOT EHR users for CHD risk” (NEUCRs) in the GRS and CRS groups was adequately powered (≥80%) for questions such as “Have you looked for any information about how genetic factors affect your chances of getting a heart attack?”, “Is there a specific internet site you like to go to for information about heart disease?”, and “Have you used the Internet to learn specifically about heart disease?” (Figure 2); comparing subjective reports of information sharing and social network between was underpowered (Figure 2). Of note however, comparing all EUCRs (n=53) to all NEUCRs (n=150) in the study was adequately powered (n≥80%) for the question, “Have you encouraged others to be screened for their CHD risk?” (OR 2.94; CI: 1.47,60.13; P=0.002). The effect size for each of several additional survey responses was described in the previous sections.

Multivariate analyses

Participants with college-level education or higher were more likely to ever log in to their EHR (OR 30.01; CI: 1.33,70.12; P=0.010) (Appendix Table A.6). Females were more likely to login 51-100 times over the course of the trial (OR 2.79; CI: 10.09,7.67; P=0.040), and those with a family history of CHD were more likely to login 101-200 times (OR 3.79; CI: 10.16,12.91; P=0.030). Thus, female sex, education level, and family history of CHD were all predictors of objective quantification of EHR login.

APPENDIX C

Expansion of Results

Reported electronic health record (EHR) access associates with internet use

At baseline, within the GRS group, EUCRs were more likely than NEUCRs to visit an internet website to learn specifically about heart disease (OR 3.65; CI: 1.29,10.69*; P*=0.02) (Appendix Figure A.2). Within the CRS group, EUCRs were more likely than NEUCRs to look for health or medical information in general (OR 6.21; CI: 10.13,116.44*; P*=0.04) (Appendix Figure A.2).

At three months post-disclosure, within the GRS group, EUCRs were more likely than NEUCRs to use the internet to look for information about how genetic factors affect CHD risk (OR 4.62; CI: 1.78,12.43*; P*=0.002), use the internet to look for information about heart disease (OR 11.3; CI: 3.3,470.14, *P*<0.0001), use the internet to look for health or medical information in general (OR 2.82; CI: 10.07,8.21*; P*=0.04), and visit an internet website to learn specifically about heart disease (OR 9.54; CI: 2.92,35.79*; P*=0.0001) (Figure 2a).

At three months post-disclosure, within the CRS group, EUCRs were more likely than NEUCRs to use the internet to look for information about how personal health habits affect CHD risk (OR 0.039; CI: 10.0,10.93*; P*=0.048), use the internet to look for information about heart disease (OR 9.49; CI: 2.78,34.7*; P*=0.0003), use the internet to look for health or medical information in general (OR 3.68; CI: 10.07,170.19*; P*=0.04), use a website to help with diet, weight, or physical activity (OR 90.15; CI: 2.89,33.88*; P*=0.0001), and visit an internet website to learn specifically about heart disease (OR 61.51; CI: 10.86,560.84, *P*<0.0001) (Figure 2b).

At six months post-disclosure, within the GRS group, EUCRs were more likely than NEUCRs to use the internet to look for information about heart disease (OR 3.3; CI: 10.16,9.68*;* *P*=0.03), visit an internet website to learn specifically about heart disease (OR 14.23; CI: 3.33,78.96*; P*=0.0002), use the internet to look for information about how personal health habits affect CHD risk (OR 2.74; CI: 10.13,6.9*; P*=0.03), and use the internet to look for information about how genetic factors affect CHD risk (OR 4.54; CI: 1.72,12.52*; P*=0.002) (Appendix Figure A.3a).

At six months post-disclosure, within the CRS group, EUCRs were more likely than NEUCRs to use the internet to look for information about how personal health habits affect CHD risk (OR 4.78; CI: 1.61,15.66*; P*=0.005) (Appendix Figure A.3b).

Reported EHR access associates with information sharing in social networks

Information sharing was not assessed at baseline. At three months post-disclosure, within the GRS group EUCRs were more likely than NEUCRs to discuss their CHD risk with their primary care provider (OR 3.42; CI: 1.13,12.88; *P*=0.03) and encourage others to be screened for their CHD risk (OR 2.95; CI: 1.16,8.14; *P*=0.02) (Figure 3a). Within the CRS group EUCRs were more likely than NEUCRs to share their CHD risk with their children (OR 3.22; CI: 1.09,10.5; *P*=0.03) at six months post-disclosure (Appendix Figure A.4d).

At three months post-disclosure, within the GRS group EUCRs were more likely than NEUCRs to have friends or family members with whom they discussed their health (OR 9.18; CI: 1.59,175.99; *P*=0.01) and have community organizations that provide them with health information (OR 9.26; CI: 1.6,117.32; *P*=0.01) (Figure 3c). At six months post-disclosure, within the GRS group EUCRs remained more likely than NEUCRs to have friends or family members

with whom they discussed their health (OR 10.59; CI: 1.86,201.79; *P*=0.005) and have community organizations that provide them with health information (OR 3.10; CI: 1.11,8.84; *P*=0.03) (Figure 3c).

Quantified EHR access associates with self-reported information exchange behaviors

Quantified EHR access was assessed. Moving from one section of the EHR to another without manually logging off did not affect the login count. Four categories of EHR access frequency were easily identifiable and were therefore used to guide subsequent analysis in the study. The most common frequency was 1-50 times (61%), and a second most common 51-100 times (15%). The EHR was accessed more than 100 times by 11% of all participants, while 15% did not access the EHR at all. GRS participants were more likely than CRS participants to access their EHR (OR 2.50; CI: 1.07,5.86; *P*=0.04) (Table 3).

To determine associations between TotalLogin and favorable survey question responses, we calculated the adjusted mean and standard error for the total number of EHR access counts (TotalLogin) for individuals who responded ‘Yes’ to each survey question. This was compared to the adjusted mean and standard error for the total number of EHR access counts (TotalLogin) for individuals who responded ‘No’ to each survey question (Appendix Table A.4).

For comparison, TotalLogin was also determined for a random sample (using random numbers generated from https://www.randomizer.org/) of approximately 200 Mayo Clinic patients aged 45-65 years, who signed up for access to their EHR but were not in the MI-GENES study. These data were not adjusted for sociodemographic characteristics, since these data were not available for individuals who were not formally enrolled in the MI-GENES study. The unadjusted mean TotalLogin for the random sample of patients not formally in the MI-GENES

study was 21(40). The most common frequencies were 1-50 (66%) and 51-100 (7%), and 4% of individuals accessed the portal more than 100 times over the course of the study, similar to MI-GENES Study participants.

At three months after CHD risk disclosure, individuals who self-reported using the EHR to access information related to their CHD risk (70(10) versus 36(5); *P*=0.002), or to communicate with their doctor's office (61(7) versus 25(6), *P*<0.0001) or keep track of personal health information (58(5) versus 15(7), *P*<0.0001), had a higher quantified EHR access frequency than those who answered no to these survey items; this persisted at six months post-disclosure (Table 2).

Those who reported using the internet to look for CHD information (64(10) versus 38(5); *P*=0.02), encouraging others to be screened for their CHD risk (51(6) versus 32(7); *P*=0.03), having friends or family members with whom they discuss health issues (46(5) versus 24(10); *P*=0.04), or having community organizations that provide them with health information (59(9) versus 38(5); *P*=0.04) at three months post-disclosure all had a higher total quantified EHR access than those who answered no to these questions (Table 2). Those who reported using the internet to look for any information about how genetic factors affect their CHD risk trended towards having a higher total quantified EHR access than those who answered no to these questions (50(7) versus 37(6); *P*=0.07).

Impact of GRS on internet use and information sharing in social networks

At three months post-disclosure, EUCRs with high GRS were more likely than NEUCRs with high GRS to share their CHD risk with family members (OR inf; CI: 3.64,inf; *P* =0.004), but were less likely than EUCRs with low GRS to share their CHD risk with their children (OR

0.17; CI: 0.03,0.81; *P*=0.03). Perhaps this finding reflects a desire to protect their children while seeking reassurance, and while alerting others to risk, given the 10% or greater increase in risk relative to the general population. Interestingly, EUCRs with low GRS were more likely than EUCRs with high GRS to encourage others to be screened for their CHD risk at three months post-disclosure (OR 3.61; CI: 1.09,13.58; *P*=0.040), possibly suggesting some degree of optimism and hesitation by low GRS and high GRS individuals, respectively.

At 6 months post-disclosure, EUCRs in the GRS group were more likely than EUCRs in the CRS group to use the internet to look for information about heart disease (OR 5.76; CI: 1.22,42.81); *P*=0.03), visit an internet web site to learn specifically about heart disease (OR 9.75; CI: 1.68,90.28; *P*=0.01), and have a specific internet site they like to visit for information about heart disease (OR 10.73; CI: 1.36,243.95; *P*=0.02) (Web Figure 3a). EUCRs with high GRS were more likely to use the internet to look for information about heart disease (OR 9.78; CI: 2.27,54.2; *P*=0.002), and visit a website to learn specifically about heart disease (OR 47.19; CI: 4.82,1222.83; *P*=0.0004), than EUCRs with low GRS (data not shown visually). Thus, GRS disclosure further increased internet use outside of the patient portal in EUCRs.

At six months post-disclosure, EUCRs in the GRS group were also more likely than EUCRs in the CRS group to have friends or family members with whom they discussed health (OR 15.74; CI: 1.54,160.97; P=0.0201) (Appendix Figure A.6b). EUCRs with high GRS were also more likely than NEUCRs with high GRS to liaise with community organizations that provide them with health information (OR 7.43; CI: 1.17,55.12; *P*=0.030).

Additional Trends

Overall quantified Electronic Health Record (EHR) access

GRS participants trended towards being more likely than CRS participants to access their EHR 51-100 times (OR 1.87; CI: 0.83,4.40; P=0.13) or 101-200 times (OR 2.95; CI: 0.87,120.14; P=0.09) (Table 3).

Internet use by EUCRs

At baseline, within the GRS group, EUCRs trended towards being more likely than NEUCRs to use the internet to look for information about heart disease (OR 40.12; CI: 0.90,22.29; P=0.07) and look for health or medical information in general (OR 2.69; CI: 0.91,9.32; P=0.08). EUCRs among the CRS participants trended towards being more likely to use a website to help with diet, weight, or physical activity (OR 2.83; CI: 0.97,8.92; P=0.06), visit an internet website to learn specifically about heart disease (OR 2.96; CI: 0.86,9.76; P=0.08), and use the internet to look for information about heart disease (OR 3.51; CI: 0.62,18.36; P=0.15).

At three months after risk disclosure, within the GRS group, EUCRs trended towards being more likely than NEUCRs to use the internet to look for information about how personal health habits affect CHD risk (OR 20.14; CI: 0.82,60.03; P=0.12), use a website to help with diet, weight, or physical activity (OR 2.29; CI: 0.95,5.66; P=0.07), and use the internet to look for information about how personal health habits can affect CHD risk (OR 20.14; CI: 0.82,60.03; P=0.12) (Appendix Figure A.3a). Within the CRS group, EUCRs were more likely than NEUCRs to use the internet to look for information about how genetic factors affect CHD risk (OR 2.93; CI: 10.0,8.9; P=0.047) (Figure 2b).

At six months after risk disclosure, within the GRS group, EUCRs trended towards being more likely than NEUCRs to use the internet to look for health or medical information in general (OR 2.5; CI: 0.94,7.33; P=0.07). EUCRs among the CRS participants trended towards being more likely to look for health or medical information in general (OR 2.68; CI: 0.85,10.28; P=0.09), use a website to help with diet, weight, or physical activity (OR 2.76; CI: 0.94,80.16; P=0.06), and visit an internet website to learn specifically about heart disease (OR 4.65; CI: 0.48,460.16; P=0.17) (Appendix Figure A.3b).

Information sharing by EUCRs

At three months after risk disclosure, within the GRS group, EUCRs trended towards being more likely than NEUCRs to share their CHD risk with others (OR 3.27; CI: 0.78,22.66; P=0.11) (Figure 3a). EUCRs among the CRS participants trended towards being more likely to share their CHD risk with their siblings (OR 2.95; CI: 0.98,90.19; P=0.06), their spouses (OR 2.98; CI: 0.74,20.26; P=0.14), and their primary care provider (OR 2.76; CI: 0.88,10.6; P=0.09), and to encourage others to be screened for their CHD risk (OR 2.59; CI: 0.89,80.05; P=0.08) (Figure 3b).

Interestingly, at three months after risk disclosure, EUCRs with high GRS trended towards being less likely than NEUCRs with high GRS to share their CHD risk specifically with their children (OR 0.34; CI: 0.1,10.07; P=0.07).

At six months after risk disclosure, within the GRS group, EUCRs trended towards being more likely than NEUCRs to share their CHD risk with others (OR 5.52; CI: 0.93,1060.08; P=0.06), particularly their friends (OR 2.37; CI: 0.95,60.01; P=0.06). Within the CRS group, EUCRs trended towards being more likely than NEUCRs to share their CHD risk with others

(OR 2.74; CI: 0.66,18.8*; P*=0.18), particularly their spouses (OR 3.48; CI: 0.86,23.69*; P*=0.09) (Appendix Figure A.3d). EUCRs with high GRS persisted in the trend towards being less likely than NEUCRs with high GRS to share their CHD risk with their children (OR 0.33; CI: 0.1,10.05*; P*=0.06).

Social network by EUCRs

At baseline, within the GRS group, EUCRs trended towards being more likely than NEUCRs to have friends or family members with whom they talked about their health (OR 40.07; CI: 0.93,290.06*; P*=0.06) and have community organizations that provide them with health information (OR 20.15; CI: 0.83,5.56*; P*=0.11) (Figure 3c). Similarly, within the CRS group, EUCRs trended towards being more likely than NEUCRs to have friends or family members with whom they talked about their health (OR 4.36; CI: 0.62,88.99*; P*=0.15) (Figure 3d).

Impact of GRS on information exchange by EUCRs

EUCRs with low GRS trended towards being more likely to share their CHD risk with extended family members (OR 4.51, CI 0.77-39.32, *P*=.1) and encourage others to be screened for their CHD risk (OR 2.71, CI 0.92-8.24, *P*=.07) at three months post-disclosure, than NEUCRs with low GRS.

At 6 months after risk disclosure, EUCRs in the GRS group trended towards being more likely to look for any information about how genetic factors affect their CHD risk (OR 30.16; CI: 0.86,130.04*; P*=0.09) than EUCRs in the CRS group (Figure 2). EUCRs in the GRS group

trended towards being less likely than EUCRs in the CRS group to share their CHD risk with their children (OR 0.42; CI: 0.11,1.44*; P*=0.17).

APPENDIX D

Evolution of EHR Use in the MI-GENES Study

Interestingly, correlation of total EHR login with between-visits time periods increased as the study progressed (Appendix Table A.7). Accordingly, total login throughout the study correlated most with the total login during the last three months of follow-up. This suggests that the snapshot captured in the total EHR login value most represents EHR access after risk disclosure. This is consistent with the correlation of EHR login (i) prior to risk disclosure with (ii) EHR login during time periods following risk disclosure, declining steadily as the study progressed.

Psychosocial factors help determine information exchange, based on various models that have been purported over several decades, and are currently being investigated in ongoing studies in our group. Additional analyses would be needed to confirm the reliability, validity, and prognostic value of the EHR access survey instrument presented in our exploratory analysis.

Limitations of Assessing EHR Access

This suggests a possible element of recall bias. In a publication by the United States Government Accountability Office reporting on national survey by The Department of Health and Human Service, patients described having trouble remembering the passwords created for their various patient portals [1]. In another study, participants did not recall that their physician had offered them access to their patient portal [2]. It may therefore not be surprising that some patients may not recall signing up for one or more of these patient portals at all if they have not frequently (or recently) used the portal. Further, in clinical practice we have observed that family members (e.g., spouses, adult children) frequently sign up for the patient portal while the patient during a clinic visit may have forgotten or reportedly be unaware. Patients who do remember signing up for the portal often give access to their family members or caregivers [3,4].

Family members (or caregivers) may be accessing the portal instead of or in addition to the patient, in which case family members or caregivers could function as influencers or spreaders of health information in their own social networks even beyond the index patients themselves. We were not able to investigate the location of each login episode, nor did we assess whether study participants may have shared their login credentials with others, such as family members, caregivers, or close friends.

APPENDIX E

Genetic versus other health information

Due to shared familial risk, an index patient may more readily share genetic risk information with others in their biological networks [5,6]. Because of the potential for communal coping and collaborative lifestyle efforts [7], the GRS information may also be shared in non-biological social networks among friends and co-workers. In addition, while GRS disclosure increases information exchange by EUCRs in their biological and non-biological social networks, we recognize that other non-traditional CHD risk factors could potentially also affect information exchange in EUCRs; the latter has not been studied. Of course, a future study could add a third group in a trial such as the MI-GENES trial, with the third group of participants receiving non-traditional CHD risk information, to address this.

It is interesting to consider that cascade screening of patients (or their family members – whether indirectly invited for screening by the patient or directly by the health care team) could be cost-effective in cardiovascular public health, as suggested by studies on familial hypercholesterolemia [8-10]. However, cascade screening has so far been used for mendelian disorders such as familial hypercholesterolemia, cannot readily be compared to polygenic disorders such as coronary heart disease.

APPENDIX F

Conceptual Model

In our conceptual model of EUCRs and NEUCRs in social networks, EUCRs (Figure 3b, purple schematic) had a high frequency of quantified EHR access via a patient portal (wide/thick dashed lines) and shared their CHD risk information with at least one of four evaluated spheres of influence (white circles with black borders), with a skew (reported by approximately one in five individuals) towards a higher number of communication ties or connections (solid black lines), i.e., sharing CHD risk information with three out of the four evaluated spheres of influence. Conversely, NEUCRs (Figure 3b, yellow schematic) had a low frequency of quantified EHR access via a patient portal (narrow/thin dashed lines); approximately one in ten NEUCRs did not share their CHD risk information in any of the spheres of influence (white circles with black borders), while a similar fraction shared their CHD risk information with three spheres of influence, with no skew towards a higher number of ties (solid black lines). Neither EUCRs nor NEUCRs tended to report sharing CHD risk information with all four spheres of influence. Betweenness centrality and other mathematical components of a formal social network analysis were not calculated. A simplified model that would be accessible to a variety of practitioners, including clinicians without training in social network analysis, was pursued.

REFERENCES

1. Health Information Technology: HHS Should Assess the Effectiveness of Its Efforts to Enhance Patient Access to and Use of Electronic Health Information. United States Government Accountability Office, 2017. Accessed September 2018, at https://www.gao.gov/products/GAO-17-305.)

2. Goel MS, Brown TL, Williams A, Cooper AJ, Hasnain-Wynia R, Baker DW. Patient reported barriers to enrolling in a patient portal. J Am Med Inform Assoc 2011;18 Suppl 1:i8-12.

3. Wolff JL, Darer JD, Larsen KL. Family Caregivers and Consumer Health Information Technology. J Gen Intern Med 2016;31:117-21.

4. Wolff JL, Darer JD, Berger A, et al. Inviting patients and care partners to read doctors' notes: OpenNotes and shared access to electronic medical records. J Am Med Inform Assoc 2017;24:e166-e72.

5. Christophe V, Vennin P, Corbeil M, Adenis C, Reich M. Social sharing of genetic information in the family: a study on hereditary breast and ovarian cancers. J Health Psychol 2009;14:855-60.

6. Sturm AC. Cardiovascular Cascade Genetic Testing: Exploring the Role of Direct Contact and Technology. Front Cardiovasc Med 2016;3:11.

7. Koehly L, Loscalzo A. Adolescent Obesity and Social Networks. Prev Chronic Dis 2009;6:A99.

8. Ademi Z, Watts GF, Pang J, et al. Cascade screening based on genetic testing is cost-effective: evidence for the implementation of models of care for familial hypercholesterolemia. J Clin Lipidol 2014;8:390-400.

9. Nherera L, Marks D, Minhas R, Thorogood M, Humphries SE. Probabilistic cost-effectiveness analysis of cascade screening for familial hypercholesterolaemia using alternative diagnostic and identification strategies. Heart 2011;97:1175-81.

10. Chen CX, Hay JW. Cost-effectiveness analysis of alternative screening and treatment strategies for heterozygous familial hypercholesterolemia in the United States. Int J Cardiol 2015;181:417-24.
